# Supplementary material for: Marker-free co-selection for successive rounds of prime editing in human cells
Source: Nat Commun. 2022 Oct 7;13:5909. doi: 10.1038/s41467-022-33669-z (PMC9546848; doi:10.1038/s41467-022-33669-z)
Supplement: Supplementary file 2 — Reporting Summary [file 41467_2022_33669_MOESM2_ESM.pdf]

nature portfolio

Corresponding author(s): Yannick Doyon

Last updated by author(s): Sep 20, 2022

Reporting Summary

Nature Portfolio wishes to improve the reproducibility of the work that we publish. This form provides structure for consistency and transparency in reporting. For further information on Nature Portfolio policies, see our Editorial Policies and the Editorial Policy Checklist.

Statistics

For all statistical analyses, confirm that the following items are present in the figure legend, table legend, main text, or Methods section.

n/a	Confirmed	
☐	☒	The exact sample size (*n*) for each experimental group/condition, given as a discrete number and unit of measurement
☐	☒	A statement on whether measurements were taken from distinct samples or whether the same sample was measured repeatedly
☒	☐	The statistical test(s) used AND whether they are one- or two-sided<br>*Only common tests should be described solely by name; describe more complex techniques in the Methods section.*
☒	☐	A description of all covariates tested
☒	☐	A description of any assumptions or corrections, such as tests of normality and adjustment for multiple comparisons
☐	☒	A full description of the statistical parameters including central tendency (e.g. means) or other basic estimates (e.g. regression coefficient) AND variation (e.g. standard deviation) or associated estimates of uncertainty (e.g. confidence intervals)
☒	☐	For null hypothesis testing, the test statistic (e.g. *F*, *t*, *r*) with confidence intervals, effect sizes, degrees of freedom and *P* value noted<br>*Give P values as exact values whenever suitable.*
☒	☐	For Bayesian analysis, information on the choice of priors and Markov chain Monte Carlo settings
☒	☐	For hierarchical and complex designs, identification of the appropriate level for tests and full reporting of outcomes
☒	☐	Estimates of effect sizes (e.g. Cohen's *d*, Pearson's *r*), indicating how they were calculated

Our web collection on statistics for biologists contains articles on many of the points above.

Software and code

Policy information about availability of computer code

Data collection	BD LSRII flow cytometer - FACSdiva v6.1.2 software.
Data analysis	BEAT (PMID: 31328964), TIDE (PMID: 29538768), TIDER (PMID: 29538768), FlowJo v10 an v11.

For manuscripts utilizing custom algorithms or software that are central to the research but not yet described in published literature, software must be made available to editors and reviewers. We strongly encourage code deposition in a community repository (e.g. GitHub). See the Nature Portfolio guidelines for submitting code & software for further information.

Data

Policy information about availability of data

All manuscripts must include a data availability statement. This statement should provide the following information, where applicable:

- Accession codes, unique identifiers, or web links for publicly available datasets
- A description of any restrictions on data availability
- For clinical datasets or third party data, please ensure that the statement adheres to our policy

Source data for the figures are provided as a Source Data file. All vectors generated in this study have been deposited to Addgene (https://www.addgene.org/browse/article/28219935/). All raw Sanger sequencing data generated in this study are available on request from the corresponding author [YD].

Field-specific reporting

Please select the one below that is the best fit for your research. If you are not sure, read the appropriate sections before making your selection.

[x] Life sciences   [ ] Behavioural & social sciences   [ ] Ecological, evolutionary & environmental sciences

For a reference copy of the document with all sections, see nature.com/documents/nr-reporting-summary-flat.pdf

Life sciences study design

All studies must disclose on these points even when the disclosure is negative.

Sample size	Sample sizes were determined based on literature precedence for genome editing experiments (PMID: 34653350, PMID: 33893286, PMID: 36008610).
Data exclusions	No data was excluded
Replication	All experiments were repeated at least once. All attempts at replication were successful and provided in the manuscript
Randomization	Cells were transfected and grown under identical conditions. No randomization was performed
Blinding	Cells were transfected and grown under identical conditions. No blinding was used

Reporting for specific materials, systems and methods

We require information from authors about some types of materials, experimental systems and methods used in many studies. Here, indicate whether each material, system or method listed is relevant to your study. If you are not sure if a list item applies to your research, read the appropriate section before selecting a response.

Materials & experimental systems

n/a	Involved in the study	
[ ]	[x]	Antibodies
[ ]	[x]	Eukaryotic cell lines
[x]	[ ]	Palaeontology and archaeology
[x]	[ ]	Animals and other organisms
[x]	[ ]	Human research participants
[x]	[ ]	Clinical data
[x]	[ ]	Dual use research of concern

Methods

n/a	Involved in the study	
[x]	[ ]	ChIP-seq
[ ]	[x]	Flow cytometry
[x]	[ ]	MRI-based neuroimaging

Antibodies

Antibodies used	anti-α-Na+/K+ ATPase antibody (Invitrogen, catalog # MA3-928).<br>anti-α-Tubulin antibody (Santa Cruz Biotechnology, catalog # sc-32293).
Validation	The anti-α-Na+/K+ ATPase antibody (Invitrogen, catalog # MA3-928) went through Advanced Verification by knockdown to ensure that the antibody binds to the antigen stated (https://www.thermofisher.com/antibody/product/ATP1A1-Antibody-clone-M7-PB-E9-Monoclonal/MA3-928). The highly cited anti-α-Tubulin antibody (Santa Cruz Biotechnology, catalog # sc-32293, cited 563 times) binds to a single specific 50 kDa band corresponding to α-Tubulin (See Supplementary Fig. 7).

Eukaryotic cell lines

Policy information about cell lines

Cell line source(s)	K562 (ATCC), U2OS (ATCC), HeLa S3 (ATCC)
Authentication	Cells were authenticated by the supplier using STR analysis.
Mycoplasma contamination	All cell lines tested negative for mycoplasma and were tested periodically.
Commonly misidentified lines (See ICLAC register)	None

Flow Cytometry

Plots

Confirm that:

- [x] The axis labels state the marker and fluorochrome used (e.g. CD4-FITC).
- [x] The axis scales are clearly visible. Include numbers along axes only for bottom left plot of group (a 'group' is an analysis of identical markers).
- [x] All plots are contour plots with outliers or pseudocolor plots.
- [x] A numerical value for number of cells or percentage (with statistics) is provided.

Methodology

Sample preparation	Cells were collected via centrifugation, washed in PSB and resuspended in PBS for analysis
Instrument	BD LSRII flow cytometer
Software	FlowJo software (Tree Star)
Cell population abundance	A minimum of 100 000 events were counted for each sample.
Gating strategy	Cells were initially gated on population using FSC and SSC

- [x] Tick this box to confirm that a figure exemplifying the gating strategy is provided in the Supplementary Information.
