# Supplementary material for: Marker-free co-selection for successive rounds of prime editing in human cells
Source: Nat Commun. 2022 Oct 7;13:5909. doi: 10.1038/s41467-022-33669-z (PMC9546848; doi:10.1038/s41467-022-33669-z)
Supplement: Supplementary file 5 — Description of Additional Supplementary Files [file 41467_2022_33669_MOESM5_ESM.pdf]

Title: Supplementary Data 1

Legend: Oligo and DNA sequences, transfection mixes, Addgene vectors
